# Impact of postoperative positioning on surgical and visual outcomes after pars plana vitrectomy for rhegmatogenous retinal detachment: a systematic review and meta-analysis

**DOI:** 10.1186/s40942-026-00890-7

**Published:** 2026-06-25

**Authors:** Yousef Mesaed Al-Shammari, Aljawhara Mesaed Al-Shammari, Mahmoud Alrabiah

**Affiliations:** 1 Department of Ophthalmology, Al- Bahar Eye Center, Ibn Sina Hospital, Ministry of Health, Kuwait City, Kuwait; 2 Faculty of Medicine, Aljowhara Mesaed Al-Shammari, Alexandria University, Alexandria, Egypt; 3 Retina Unit, Al-Adan Hospital, Kuwait City, Kuwait; 4 Retina Team, Al-Bahar Eye Center and Alrabiah Medical Center, Kuwait City, Kuwait

**Keywords:** Pars plana vitrectomy, Rhegmatogenous retinal detachment, Prone, Supine, Review

## Abstract

**Introduction:**

Rhegmatogenous retinal detachment (RRD) is a serious ophthalmic pathology managed with pars plana vitrectomy (PPV) with gas tamponade. Even though prone position after surgery is widely used, it has been questioned because of high patient discomfort and contradictory evidence concerning its effects on functional outcomes.

**Objective:**

To assess the effect of various positioning options post-surgery on the rate of retinal reattachment and visual outcome after PPV for RRD.

**Methods:**

A systematic review and meta-analysis were carried out in accordance with the PRISMA statement. Searches for related papers in PubMed, Scopus, and Web of Science databases were made up to April 2026. Articles describing postoperative positioning after PPV for RRD were considered for inclusion. Primary outcomes were retinal reattachment rate and changes in best corrected visual acuity (BCVA).

**Results:**

Ten studies with 1262 eyes were included. Pooled analysis showed no statistically significant difference in retinal reattachment rate between prone/face-down positioning and support-the-break/adjustable positioning (RR = 0.96, 95% CI 0.84 to 1.11, *p* = 0.231), with moderate heterogeneity. The evidence for reattachment rate outcome was of low certainty. Two studies with 90 eyes showed a very small borderline difference in logMAR BCVA change in favour of prone positioning versus supine positioning (MD = -0.07, 95% CI -0.14 to 0.00, *p* = 0.049). However, the certainty of evidence for this comparison was very low. The clinical relevance of this small difference is uncertain. Descriptive single-arm analyses demonstrated postoperative improvement in BCVA for both the prone and supine positioning arms, nonetheless, there was significant heterogeneity in these analyses.

**Conclusion:**

In the pooled analysis, there was no statistically significant difference in the rate of retinal reattachment between prone positioning and support-the-break positioning after PPV for RRD, and the certainty of evidence for this outcome was low. For BCVA, the pooled analysis showed a small borderline difference in logMAR BCVA in favour of prone positioning versus supine positioning. However, the certainty of evidence for this comparison was very low and the clinical importance of this finding is unclear. Thus, the current evidence does not allow a strong conclusion of clinically meaningful functional superiority of prone positioning. Postoperative positioning should be determined by the location of the retinal break, the tamponade agent, surgical factors, patient tolerance, and the judgment of the clinician.

**Clinical trial number:**

Not applicable.

**Supplementary Information:**

The online version contains supplementary material available at https://doi.org/10.1186/s40942-026-00890-7.

## Introduction

Rhegmatogenous retinal detachment (RRD) is a serious acute condition that may lead to irreversible visual loss without adequate treatment [1]. Today, pars plana vitrectomy (PPV) with gas tamponade is one of the most common procedures used for RRD treatment in developed countries because of advances in instrument sizes, wide-angle visualization, and improvements in surgical technique [2]. As a result, single-surgery anatomical success rates approaching or exceeding 90% have been reported in many contemporary series [3].

Nonetheless, there continue to be problems with visual outcome after RRD treatment, especially when the macula is involved. Macula-involving rhegmatogenous RD repair is characterized by a high anatomical success rate; however, in most cases, postoperative distortion occurs (67% – 89%) and may negatively affect patients’ quality of life [4]. Besides anatomical reattachment, retinal displacement, metamorphopsia, binocular diplopia, and quality of visual recovery are increasingly recognized as clinically relevant postoperative outcomes after PPV [5].

Postoperative positioning is a well-established method used in managing RRDs. The rationale for postoperative positioning is based on the physical behavior of intraocular tamponade agents: gas rises within the vitreous cavity, and the patient’s head position determines whether the gas–fluid interface adequately tamponades the causative retinal break until chorioretinal adhesion develops after retinopexy [6]. According to current recommendations, prone positioning should be used after PPV with intraocular gas tamponade to achieve optimal results concerning reattachment and tamponade efficacy (3). However, maintenance of this position is a major complaint in patients who undergo vitreoretinal surgeries and is characterized by low compliance [7]. Moreover, such positioning requirements may cause severe physical discomfort and, rarely, complications such as thrombophlebitis, ulnar nerve palsies, and pulmonary embolism [8].

Because of these concerns, alternative postoperative positioning strategies, including supine positioning, support-the-break positioning, free positioning, and adjustable positioning protocols, have been investigated. Nevertheless, there is still no consensus regarding the optimal postoperative positioning strategy after PPV for RRD repair. On the one hand, some data show high success rates without strict postoperative positioning requirements [9]; on the other hand, specific postoperative positioning strategies might affect various parameters, such as retinal displacement and postoperative complications. Retinal displacement is thought to occur when the detached neurosensory retina shifts relative to the retinal pigment epithelium during postoperative reattachment, potentially influenced by residual subretinal fluid dynamics, gravity, tamponade type, and early postoperative head position [10].

It is also unclear how different positioning strategies may influence postoperative anatomical and visual success. There have been many attempts to compare various positioning strategies; however, findings of such studies remain inconsistent, particularly across clinically distinct subgroups such as eyes with inferior retinal breaks or macula-involving detachments. Thus, further evidence synthesis is needed. Therefore, this systematic review and meta-analysis aimed to assess the effects of postoperative positioning strategies on anatomical and visual outcomes after PPV for RRD.

## Methods

The current systematic review and meta-analysis followed the PRISMA (Preferred Reporting Items for Systematic Reviews and Meta-Analyses) guidelines [11]. This systematic review and meta-analyses was registered in PROSPERO under the registration number (CRD420261402409).

### Search strategy and eligibility criteria

We systematically searched PubMed, Scopus, and Web of Science (WOS) from their inception until April 2026 for published studies evaluating the effect of postoperative positioning following pars plana vitrectomy (PPV) for rhegmatogenous retinal detachment (RRD). The search strategy included combinations of the following keywords and MeSH terms: “rhegmatogenous retinal detachment,” “pars plana vitrectomy,” “postoperative positioning,” “prone positioning,” “supine positioning,” “support-the-break positioning,” and “visual acuity.” Boolean operators (“AND” and “OR”) were used to combine search terms appropriately. Table S1.

Studies that met the inclusion criteria were randomized controlled trials, prospective or retrospective comparative studies, cohort studies, and interventional case series on the postoperative position strategies used after PPV for RRD. Either comparative postoperative position strategy studies or those that reported on postoperative outcomes resulting from a specific postoperative position strategy would qualify for inclusion in the study. There were no language restrictions. The reference list of the selected studies and relevant review articles was also manually searched for more eligible studies.

Studies were not considered for inclusion if they were review papers, meta-analysis papers, conference abstracts, editorials, letters to the editor, animal studies, or studies with insufficient information about postoperative outcomes. In cases where studies did not involve rhegmatogenous retinal detachment and/or did not provide clear postoperative outcomes associated with this type of retinal detachment, the study was excluded.

### Study selection and data extraction

Two reviewers separately conducted study selection using titles and abstracts. Full texts were reviewed for further assessment if any disagreement arose. In case of disagreement, consultation with another reviewer led to agreement. Two reviewers independently extracted the data from each article using a pre-designed Excel spreadsheet. Extracted data included study design, mean age, percentage of female participants, number of eyes, baseline best-corrected visual acuity (BCVA), intraocular pressure (IOP), and type of postoperative positioning. Outcome measures in our meta-analysis included retinal reattachment rates when comparing prone positioning against support-the-break positioning. Also, change in BCVA when comparing prone versus supine positioning, changes in BCVA when considering only the prone group, and changes in BCVA for the supine group were used. BCVA refers to the best visual acuity achievable with adequate correction, while retinal reattachment rate is the proportion of cases where there was reattachment of retina after surgery.

For studies with medians, we estimated standard deviations using the method described by Wan et al. (2014). In addition, whenever needed, we calculated standard deviations using the formula recommended by Cochrane Handbook.

### Quality assessment and certainty of evidence

Quality of evidence of the included studies was assessed using validated scales. Randomized controlled trials were assessed for the risk of bias using the Cochrane RoB-2 tool, while other study designs were evaluated with Newcastle-Ottawa scale (NOS). Two reviewers independently evaluated each included study, and any discrepancies were solved through discussion.

The certainty of evidence for each pooled outcome was assessed using the Grading of Recommendations Assessment, Development and Evaluation (GRADE) framework. The certainty of evidence was evaluated across the domains of risk of bias, inconsistency, indirectness, imprecision, and publication bias and was categorized as high, moderate, low, or very low certainty.

### Data synthesis and analysis

Pooling was done using the random-effects model to account for possible heterogeneity among studies. All statistical tests were done using R version 4.1.2 along with its metafor package version 4.8-0. Effect sizes of dichotomous variables were presented as risk ratios (RR) with their 95% confidence interval (CI), whereas effect sizes for continuous variables were expressed in terms of mean difference (MD). P-values of less than 0.05 were regarded as statistically significant. The heterogeneity was evaluated using the I² statistic, and the heterogeneity between studies was indicated if the I² exceeded 50%. Sensitivity analysis was carried out using leave-one-out method.

## Results

### Literature search results

The initial database search yielded a total of 428 records from PubMed (*n* = 152), Scopus (*n* = 184) and Web of Science (*n* = 92). After removal of 132 duplicates, 296 records screened by title and abstract. Of these, 238 records were excluded and 58 reports were requested for retrieval. All 58 reports were obtained and assessed for full-text eligibility.

**Fig. 1:**
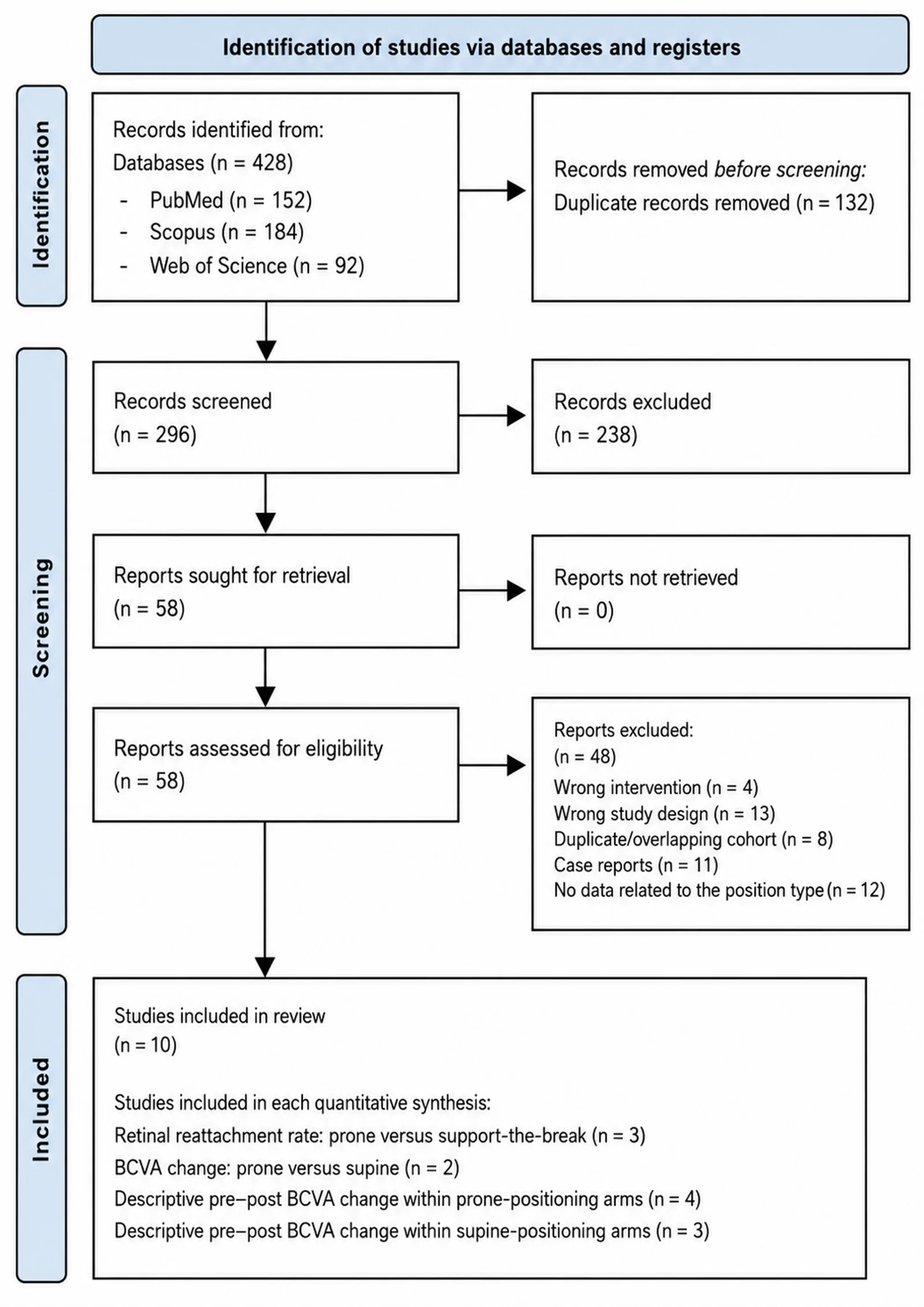
PRISMA flow chart of the selection process

After full-text evaluation, 48 reports were excluded for the following reasons: wrong intervention (*n* = 4), wrong study design (*n* = 13), duplicate or overlapping cohort (*n* = 8), case reports (*n* = 11) and no data related to position type (*n* = 12). Finally, ten studies were included in the systematic review. The studies included in the quantitative syntheses were distributed by assessed outcomes as follows: retinal reattachment rate in prone versus support-the-break positioning (*n* = 3), change in BCVA in prone versus supine positioning (*n* = 2), Descriptive pre–post change in BCVA within prone-positioning arms (*n* = 4), Descriptive pre–post change in BCVA within supine-positioning arms (*n* = 3), and studies that did not provide compatible comparator groups, outcome definitions, or extractable numerical data were summarized narratively only (*n* = 3). Table S2 shows studies contributing to each quantitative and qualitative synthesis. The entire selection process for the study is shown in Fig. 1.

### Characteristics of the included studies

Our review included ten studies, published between 2015 and 2024, comprising a total of 1,262 eyes [1, 12–20]. Studies were conducted in China (*n* = 3), Japan (*n* = 2), Egypt (*n* = 1), United Kingdom (*n* = 1), Switzerland (*n* = 1), United States (*n* = 1) and Netherlands/Italy (*n* = 1). The study designs included four randomized controlled trials, two retrospective cohort studies, two prospective non-randomized comparative studies, one prospective interventional case series and one retrospective case series.

Mean participant age varied between 38.7 and 69.3 years. The proportion of female participants reported varied between 28.0 and 51.1%. Sample sizes varied considerably across studies from 32 to 403 eyes. The number of eyes in the comparison studies ranged from 29 to 120. Baseline visual acuity also varied across studies, with values reported across studies ranging from approximately 0.26 to 1.72 logMAR, although not all studies reported BCVA specifically. Baseline estimated intraocular pressure ranged from ~ 10.0 to ~ 14.5 mmHg.

The studies included were clinically heterogeneous. Some studies included macula-off or fovea-involving rhegmatogenous retinal detachment, while others included both macula-on and macula-off. The proportion of inferior retinal breaks also varied between studies, from a small minority of cases to studies restricted to inferior or peripheral breaks. Tamponade agents included sulphur hexafluoride gas, perfluropropane gas, silicone oil or mixed gas/silicone oil protocols.

Postoperative positioning strategies were variable and included prone or face-down positioning, supine or face-up positioning, support-the-break or adjustable positioning, free or no-prone positioning, and log-roll positioning. Time of positioning varied from short immediate postoperative procedures of 5–24 h to longer procedures of 7–14 days, depending on study design, tamponade agent, and location of the retinal break.

Anatomical outcomes were also defined differently in different studies and included primary retinal reattachment, final retinal reattachment, single-surgery anatomical success, retinal displacement on fundus autofluorescence, macular shift, retinal folds, and ellipsoid zone dropout on optical coherence tomography. Visual outcomes were most reported as postoperative BCVA or change in BCVA, although some studies reported UCVA or other functional outcomes such as metamorphopsia, distortion scores, or vision-related quality of life measures. The characteristics of the included studies are summarized in Table 1_**(a−b)**_ and the operational definitions of the postoperative positioning strategies are shown in Table 2.

**Table 1a:** Baseline characteristics of included studies

Study ID	Country	Study design	Mean age, years	Female, %	Number of eyes	Baseline VA	Baseline IOP, mmHg	Macular status	Inferior breaks
Abdelkader et al. 2020	Egypt	Prospective case series	38.7	NR	Supine: 32	1.65 ± 0.71 logMAR	10.38 ± 1.8	On: 2; Off: 30	32/32
Casswell et al. 2020	UK	RCT	Prone: 60.3 ± 11.1; STB: 61.3 ± 8.2	Prone: 30.3; STB: 26.7	Prone: 119; STB: 120	3/60 median	NR	Fovea-involving: 239	NR
Peiretti et al. 2017	Netherlands	RCT	63; 65; 63; 61	32.1	14; 14; 14; 14	1.39; 1.56; 1.38; 1.54 logMAR	NR	Off: 56/56	NR
Liang et al. 2023	China	RCT	Adjustable: 55.09 ± 9.98; Free: 53.53 ± 11.58	Adjustable: 51.1; Free: 44.7	Adjustable: 47; Free: 47	Adjustable: 1.30 ± 1.02; Free: 1.65 ± 1.00	Adjustable: 12.17 ± 3.41; Free: 12.45 ± 3.48	Off: 34/47; 40/47	Adjustable: 9/47; Free: 3/47
Schawkat et al. 2019	Switzerland	RCT	69.3 ± 12.5	28	50	NR	NR	Off: 50/50	NR
Lin et al. 2017	China	Retrospective cohort	55.0 ± 13.9	44.5	403	UCVA: 1.72 ± 0.97	10.8 ± 4.1	On/off: 204/199	106/403
Chen et al. 2015	China	Prospective cohort	Prone: 52.0 ± 11.3; Adjustable: 53.8 ± 11.7	Prone: 41.4; Adjustable: 43.6	Prone: 29; Adjustable: 39	Prone: 1.40 ± 0.38; Adjustable: 1.51 ± 0.45	Prone: 11.07 ± 3.97; Adjustable: 10.03 ± 3.05	Off: 68/68	Prone: 9/29; Adjustable: 8/39
Shiraki et al. 2018	Japan	Retrospective cohort	Prone: 59.7 ± 11.8; No-prone: 60.3 ± 11.4	Prone: 32.3; No-prone: 39.0	Prone: 65; No-prone: 77	Prone: 0.6 ± 0.7; No-prone: 0.5 ± 0.8	NR	Off: 66/142	29/142
Otsuka et al. 2017	Japan	Prospective cohort	Prone: 56 ± 9; Supine: 60 ± 11	Prone: 50.0; Supine: 36.7	Prone: 32; Supine: 30	Prone: 0.26 ± 0.52; Supine: 0.46 ± 0.62	Prone: 14.5 ± 2.9; Supine: 14.5 ± 2.6	Off: 15/32; 14/30	Prone: 12/32; Supine: 11/30
Babel et al. 2024	USA	Retrospective case series	63.4 ± 11.8	37.9	No-prone: 116	1.5 ± 1.1 logMAR	NR	On: 33; Off: 83	5/116

(Peiretti et al. 2017 groups: Prone without PFCO; prone with PFCO; supine without PFCO; supine with PFCO)

**Table 1b:** Surgical details, positioning strategy, follow-up, and outcomes

Study ID	Tamponade agent	Postoperative positioning	Duration of positioning	Follow-up duration	Anatomical outcome definition	Visual outcome definition
Abdelkader et al. 2020	Silicone oil	Supine	10–14 days	Oil removal 3–6 months; then 6 months	Retinal attachment after PPV and silicone oil; maintained attachment after oil removal	BCVA change in logMAR
Casswell et al. 2020	Gas	Prone vs. STB	24 h; then STB 6 days	6 months	Retinal displacement on FAF at 6 months; redetachment and retinal folds also reported	ETDRS BCVA; D-chart distortion; NEI VFQ
Peiretti et al. 2017	20% SF6	Prone vs. supine ± PFCO	5 h	3 months	Outer retinal folds, inner retinal folds, and EZ dropout on SD-OCT	BCVA change in logMAR; Amsler metamorphopsia
Liang et al. 2023	Silicone oil or C3F8	Adjustable vs. free	NR	3 months	Initial retinal reattachment after primary PPV; final reattachment after reoperation	Final BCVA in logMAR
Schawkat et al. 2019	SF6 or C3F8	Log roll vs. supine	6 h; final posture ~ 7 days	3–6 weeks	Macular shift on FAF; displacement measured in degrees	BCVA in logMAR; Amsler metamorphopsia
Lin et al. 2017	Silicone oil or C2F6/C3F8	Adjustable by break location	Gas: 1–2 weeks; SO: 3 months	22.7 ± 21.3 months	Primary retinal reattachment after initial surgery; final reattachment after second surgery	UCVA in logMAR; BCVA not reported
Chen et al. 2015	14% C3F8	Prone vs. adjustable	Prone ≥ 16 h/day ≥ 7 days	3 months	Retinal attachment at day 1, day 7, 2 weeks, and 4 weeks; final reattachment after reoperation	BCVA in logMAR at 3 months
Shiraki et al. 2018	20% SF6	Prone vs. no-prone	NR	Mean 8.5 months	Initial and final retinal reattachment; subgroup analysis for inferior breaks	BCVA in logMAR at 3 months
Otsuka et al. 2017	20% SF6	Prone vs. supine	Prone ≥ 8 days; supine ≥ 7 days	≥ 6 months	Initial and final anatomical success; redetachment, macular pucker, and retinal folds reported	BCVA at 1, 3, and 6 months
Babel et al. 2024	C3F8; SF6; silicone oil	No prone	None	Median 10 months	Single-surgery anatomical success; permanent reattachment after one operation, excluding oil removal	Preoperative to postoperative BCVA change

(Abbreviations: ACD, anterior chamber depth; BCVA, best-corrected visual acuity; C3F8, perfluoropropane; EZ, ellipsoid zone; FAF, fundus autofluorescence; IOP, intraocular pressure; LT, lens thickness; NR, not reported; OCT, optical coherence tomography; PFCO, perfluorocarbon liquid; RCT, randomized controlled trial; SF6, sulfur hexafluoride; SO, silicone oil; STB, support-the-break; UCVA, uncorrected visual acuity; VA, visual acuity)

**Table 2:** Operational definitions of postoperative positioning strategies

Study ID	Position	Operational definition / practical meaning
Abdelkader et al. 2020	Supine	Face-up positioning after PPV with silicone oil tamponade, intended to support peripheral and inferior breaks while avoiding face-down posture.
Casswell et al. 2020	Prone	Face-down positioning after PPV and gas tamponade.
	Support-the-break	Individualized positioning selected according to retinal break location to keep the gas bubble supporting the retinal break.
Peiretti et al. 2017	Prone	Face-down positioning after PPV.
	Supine	Face-up positioning after PPV.
Liang et al. 2023	Free	No strict postoperative posture; patients were allowed to choose their preferred position.
	Support-the-break	Adjustable positioning according to retinal break location; categorized as support-the-break for consistency with Table 1.
Schawkat et al. 2019	Log roll	Alternating posture consisting of 30 min face-to-temporal followed by 30 min face-down before final positioning.
	Supine	Patients lay flat on their back before moving to the final break-directed position.
Lin et al. 2017	Prone	Face-down positioning, particularly used for inferior retinal breaks.
	Support-the-break	Adjustable positioning according to break location; superior/lateral breaks allowed face-down or lateral decubitus positioning.
Chen et al. 2015	Prone	Strict face-down positioning after PPV and gas tamponade.
	Support-the-break	Adjustable positioning using upright or lateral recumbent positions according to retinal break location.
Shiraki et al. 2018	Free	No strict prone positioning; supine and lateral positioning were permitted according to break location.
	Prone	Postoperative face-down positioning after PPV with gas tamponade.
Otsuka et al. 2017	Prone	Strict prone positioning for most of the day after surgery.
	Supine	Prone positioning on surgery day, then supine positioning from postoperative day 1.
Babel et al. 2024	Free	No prescribed postoperative prone or directed positioning after PPV.

### Quality assessment and certainty of evidence

**Fig. 2:**
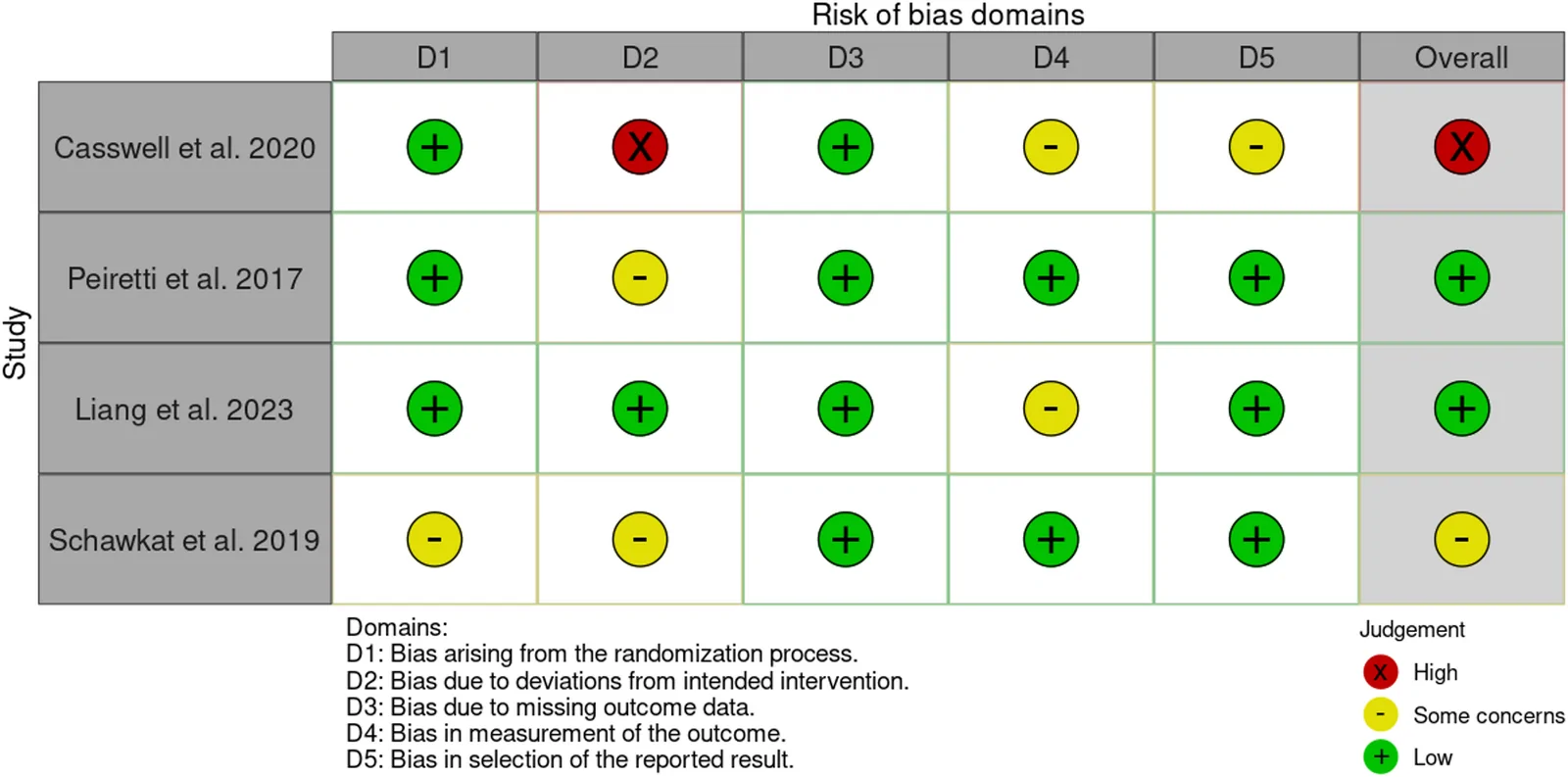
Quality assessment using RoB-2 method

Methodological quality of the included studies was evaluated using the Cochrane Risk of Bias-2 (RoB-2) criteria for randomized controlled trials (RCTs) and the Newcastle-Ottawa scale (NOS) for non-randomized studies. Among the four RCTs, one study had a high risk of bias, two had low risk of bias, and one study was judged to have some concerns with their bias potential. For more information regarding the RoB-2 assessment, check out Fig. 2.

As far as non-RCTs, NOS scores varied between 5 and 7 points out of 9 possible. Thus, all non-RCTs have been rated as either of moderate or good methodological quality, and the former is more common for included non-RCTs – 5 studies out of 6 were rated that way. Good (7/9) methodological quality has been achieved by only one non-RCT. Possible causes for such scores include non-RCT design, lack of group comparability, retrospective data collection, and, in some cases, the absence of a control group. For more information regarding the NOS assessment, check out Table 3.

**Table 3:** Quality Assessment Using Newcastle–Ottawa Scale (NOS)

Study ID	Selection (/4)	Comparability (/2)	Outcome (/3)	Total Score (/9)	Quality
Lin et al. 2017	3	1	2	6	Moderate
Chen et al. 2015	3	0	2	5	Moderate
Shiraki et al. 2018	4	1	2	7	Good
Otsuka et al. 2017	3	1	2	6	Moderate
Abdelkader et al. 2020	3	0	2	5	Moderate
Babel et al. 2024	3	0	3	6	Moderate

Assessment of GRADE indicated low certainty of evidence regarding the reattachment rate with respect to prone position compared with support-the-break position, primarily owing to the presence of high heterogeneity and imprecision. Regarding the change in BCVA with respect to prone positioning vs. supine positioning, the certainty of evidence was assessed as being very low because of the small number of studies, small sample sizes, indirectness, and imprecision. Hence, even though there was a statistically significant advantage of prone positioning regarding BCVA, this should be treated with caution Table S3.

### Meta-analysis

#### Retinal reattachment rate in prone versus support-the-break

**Fig. 3:**
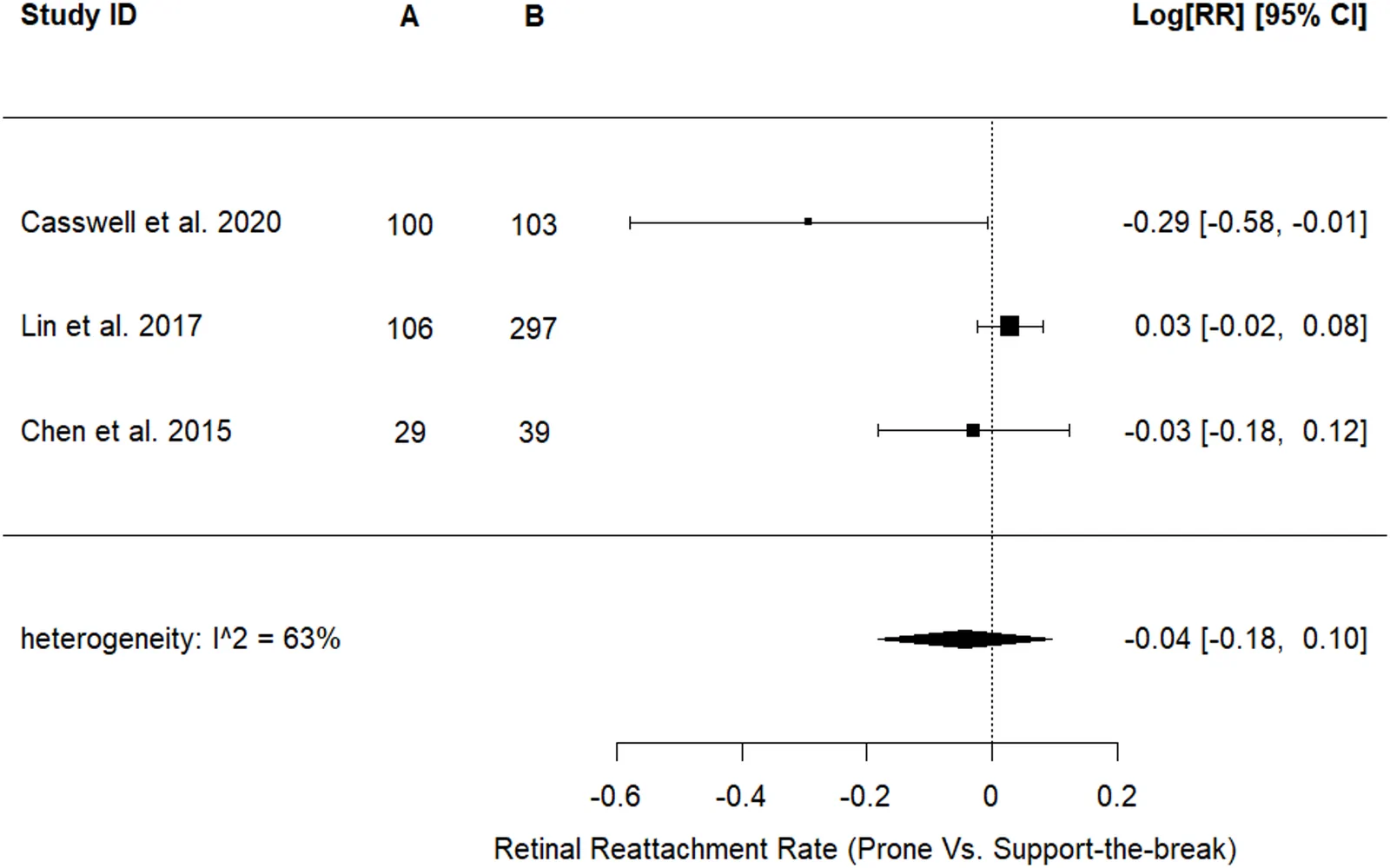
Retinal reattachment rate in prone versus support-the-break. (Group A = prone positioning; Group B = support-the-break positioning). Values less than 1 favor support-the-break positioning, whereas values greater than 1 favor prone positioning

The analysis of three including 674 eyes revealed that no statistically significant difference was found between prone and support-the-break postoperative positioning in retinal reattachment rate for eyes undergoing pars plana vitrectomy for rhegmatogenous retinal detachment (Fig. 3: RR = 0.96, 95% CI [0.84; 1.11], *P* = 0.231). A high heterogeneity was detected across the included studies (tau^2^ = 0.43, I^2^ = 63.32%). The sensitivity analysis using the leave1out method revealed that the exclusion of Casswell et al. 2020 significantly reduced the heterogeneity (I^2^ = 23.5%). Therefore, the absence of a statistically significant difference in reattachment rates should be interpreted as no clear difference in the available evidence, rather than as proof of equivalence between postoperative positioning strategies.

#### Change in best corrected visual acuity (BCVA) in prone versus supine

**Fig. 4:**
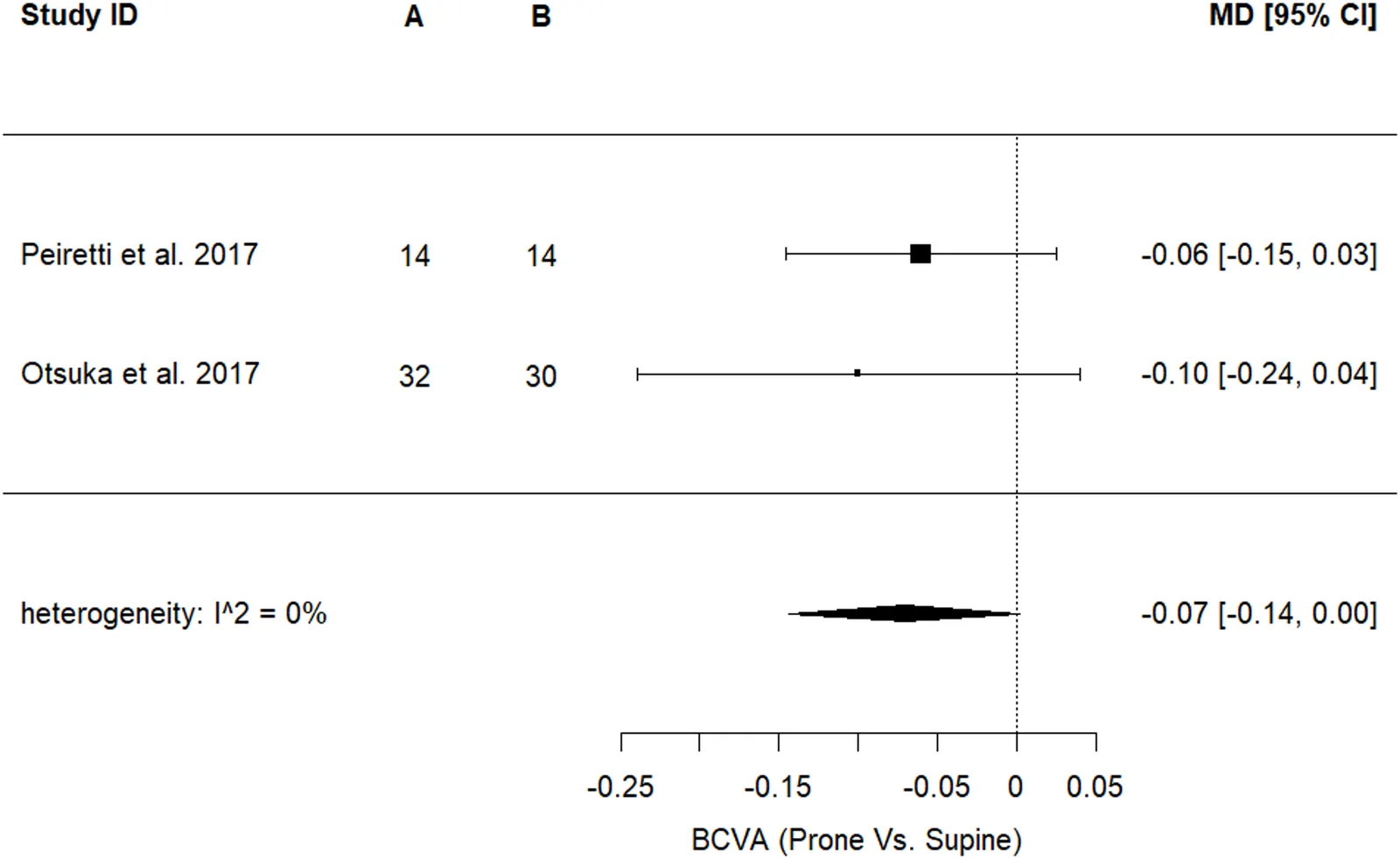
Change in best corrected visual acuity (BCVA) in prone versus supine. (Group A = prone positioning; Group B = supine positioning.) Negative mean differences favor prone positioning

The analysis of two studies including 90 eyes revealed that eyes that were placed in the prone positioning after undergoing pars plana vitrectomy for rhegmatogenous retinal detachment showed a significantly smaller change in BCVA compared to those in the supine positioning (Fig. 4: MD = -0.07, 95% CI [-0.14; 0], *P* = 0.049). Because lower logMAR values indicate better visual acuity, the negative mean difference favored prone positioning. Very low heterogeneity was observed across the included studies (τ² = 0, I² = 0%). However, only two studies were included in the analysis, the pooled effect size was very small, the p value was borderline, and the certainty of evidence was very low. Therefore, this finding should not be considered evidence of clinically meaningful functional superiority of prone compared to supine positioning.

#### Descriptive pre–post change in BCVA within prone-positioning arms

**Fig. 5:**
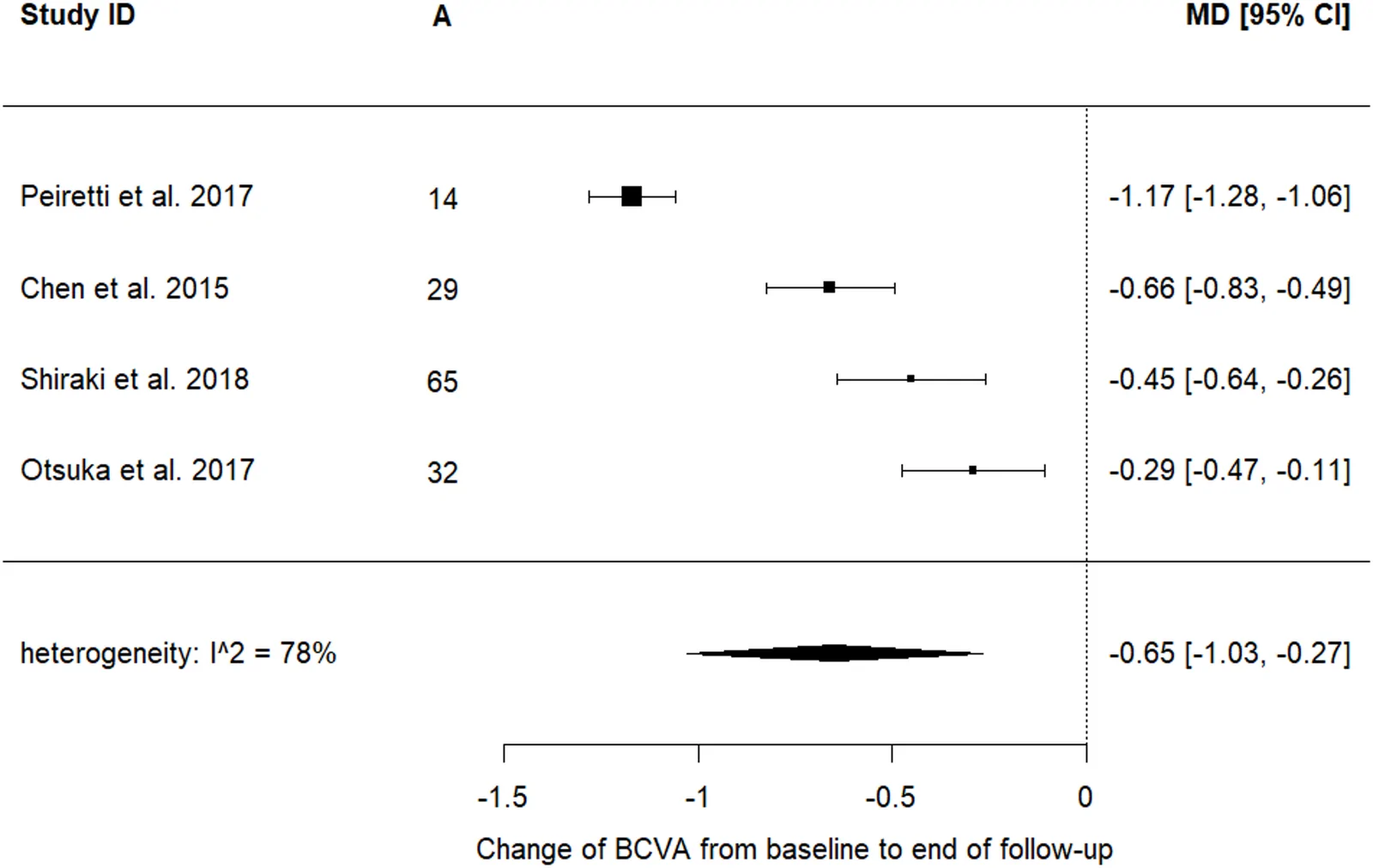
Descriptive pre–post change in best-corrected visual acuity within prone-positioning arms. (Group A = prone positioning)

The analysis of four studies including 140 eyes revealed that eyes that were placed in the prone positioning after undergoing pars plana vitrectomy for rhegmatogenous retinal detachment had a significantly decreased logMAR BCVA, indicating visual improvement postoperatively (Fig. 5: -0.65, 95% CI [-1.03; -0.27], *P* < 0.0001). A high heterogeneity was detected across the included studies (tau^2^ = 3.4, I^2^ = 77.89%). The sensitivity analysis using the leave1out method revealed no significant differences when excluding any of the included studies.

#### Descriptive pre–post change in BCVA within supine-positioning arms

**Fig. 6:**
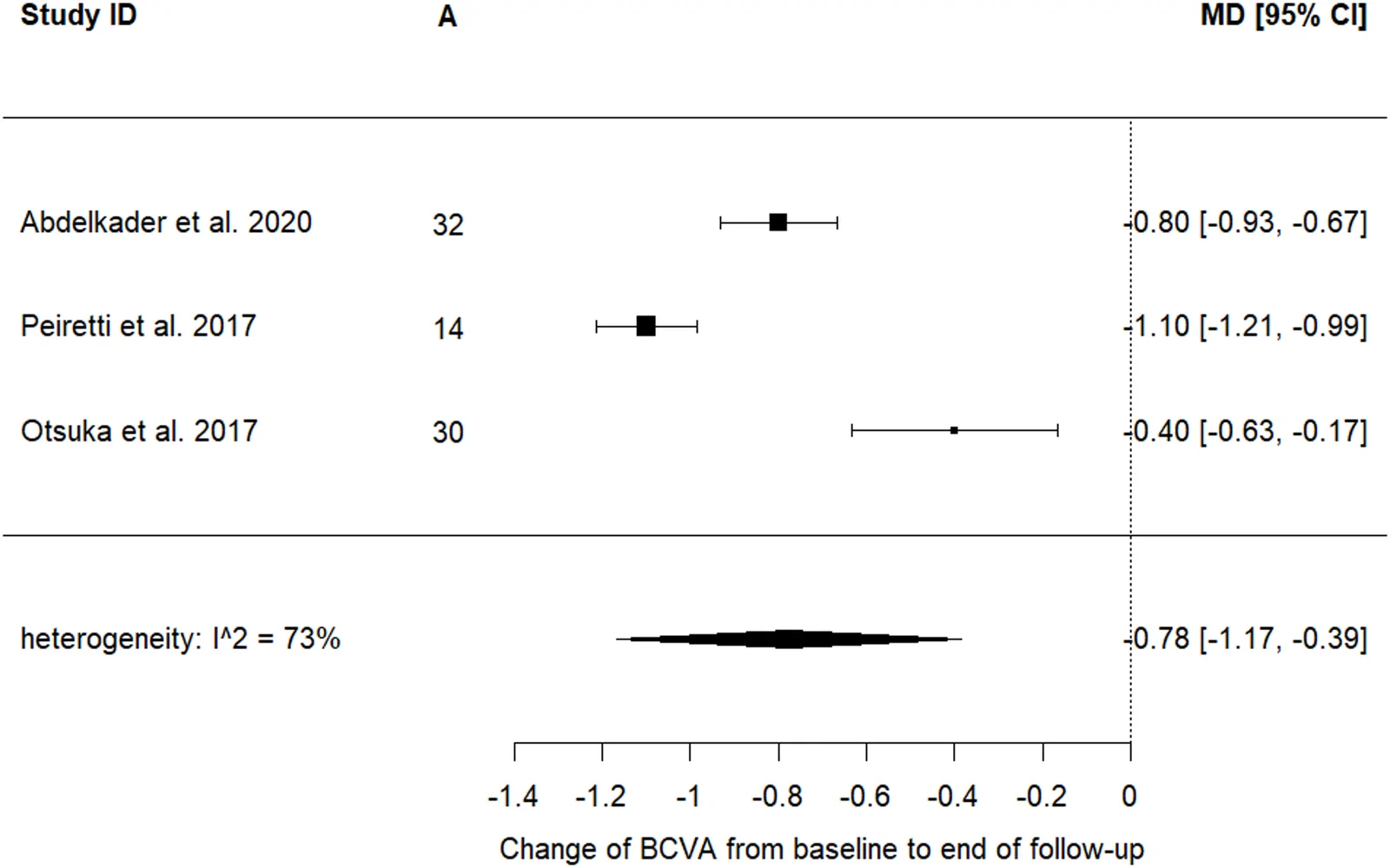
Descriptive pre–post change in best-corrected visual acuity within supine-positioning arms. (Group A = Supine positioning)

The analysis of three studies including 76 eyes revealed that eyes that were placed in the supine positioning after undergoing pars plana vitrectomy for rhegmatogenous retinal detachment had a significantly decreased logMAR BCVA, indicating visual improvement postoperatively (Fig. 6: -0.76, 95% CI [-1.17; -0.39], *P* < 0.0001). A high heterogeneity was detected across the included studies (tau^2^ = 2.6, I^2^ = 73.39%). The sensitivity analysis using the leave1out method revealed no significant differences when excluding any of the included studies.

### Systematic review

#### Retinal reattachment rate

The number of studies reporting retinal reattachment rate was five. Casswell et al. (2020) found no differences between face-down and support-the-break positioning (87.8% vs. 86.3%) [14]; Liang et al. (2023) revealed that adjustable and free positioning led to equivalent outcomes (97.9% vs. 95.7%) [19]; Chen et al. (2015) demonstrated similar success of strict face-down positioning versus adjustable positioning (89.7% vs. 92.3%) (1); Otsuka et al. (2017) proved that prolonged prone positioning had the same effect on reattachment rate as early transition into the supine positioning (93.8% vs. 93.3%) [13]; and Lin et al. (2017) concluded that high rates of reattachment could be observed using adjustable positioning according to break localization (face-down, lateral, and upright) [16]. Table 4.

**Table 4:** Retinal reattachment rate

Study ID	Arms (Positioning Strategy)	Result
Casswell et al. 2020	Face-down vs. Support-the-break	87.8% vs. 86.3%, no significant difference
Liang et al. 2023	Adjustable positioning vs. Free positioning	97.9% vs. 95.7%, no significant difference
Chen et al. 2015	Strict face-down vs. Adjustable positioning	89.7% vs. 92.3% (*P* = 1.00)
Otsuka et al. 2017	Prolonged prone vs. Early supine positioning	93.8% vs. 93.3% (*P* = 1.00)
Lin et al. 2017	Adjustable positioning	Overall 93.3% (range 92.3–95.8%), no significant difference

#### Best-Corrected Visual Acuity (BCVA)

There were ten studies analyzing BCVA among which 4 were involved in the meta-analysis while the remaining 6 studies reported non-pooled data. Casswell et al. (2020) revealed no differences between face-down and support-the-break positioning (74 letters vs. 75 letters) [14]. Peiretti et al. (2017) found similar results among four groups, namely prone or supine positioning with or without PFOC liquid [12]. Liang et al. (2023) demonstrated no differences between BCVA obtained under adjustable positioning and under the free positioning [19]. Chen et al. (2015) revealed no differences between strict face-down positioning and adjustable positioning (0.74 ± 0.25 vs. 0.77 ± 0.36 logMAR, *P* = 0.41) (1). Shiraki et al. (2018) showed that there was no difference between prone positioning and no-prone positioning [15]. Finally, Otsuka et al. (2017) indicated that prolonged prone positioning did not have an impact on BCVA when compared to early transition to the supine positioning [13]. Despite some improvements in the patients’ visual acuity postoperatively, the type of postoperative positioning did not play a role in obtaining these results.

## Discussion

This systematic review and meta-analysis evaluated the impact of postoperative positioning on surgical and visual outcomes following pars plana vitrectomy (PPV) for rhegmatogenous retinal detachment (RRD). By synthesizing data from 10 studies comprising 1,262 eyes, the present analysis provides a comprehensive and critical appraisal of the role of postoperative positioning strategies.

The principal conclusions drawn from this meta-analysis are that the choice of postoperative positioning does not make a substantial difference to surgical success, but its effect on visual results remains controversial.

First, the difference in retinal reattachment rate for prone versus support-the-break positioning groups was not statistically significant (RR = 0.96; 95% CI [0.84–1.11]). These results corresponded to data presented in some included and other sources that claimed high success rates of anatomic outcome achieved by means of modern-day PPV technology and were independent of positioning approach. Additionally, randomized and observational trials included in this analysis showed equal results regarding reattachment rates among prone, supine, and adjustable positioning strategies [12–19]. Second, concerning visual outcomes, there is evidence of a statistically significant but extremely small benefit in favor of prone position compared to supine position, concerning changes in logMAR BCVA scores (MD = − 0.07 logMAR, *p* = 0.049). Considering that the lower the logMAR score, the better the visual acuity, this negative meaning implies slightly superior visual outcomes among patients who underwent prone positioning. Nevertheless, one should exercise caution by interpreting this finding. The magnitude of the difference detected is extremely small and may not even possess clinical importance, as it might easily be within the test-retest error margin in measuring visual acuity.

There was no statistically significant difference in the rates of retinal reattachment, which suggests that in the evidence available postoperative positioning may not be the sole determinant of anatomical success after contemporary pars plana vitrectomy. However, this finding should not be interpreted as evidence of equivalence of positioning strategies. Many factors are likely to influence anatomical success, such as the surgical technique, the adequacy of vitreous base shaving, retinopexy, the tamponade agent, the location of the retinal break, the status of the macula and postoperative adherence. This conclusion is evidenced by numerous studies presented which report high success rates despite free positioning, adjustable positioning, or lack thereof [19]. Moreover, in research that deals specifically with patients who have inferior retinal breaks, it has been reported that non-prone positioning may yield equivalent or even better results [15]. However, the results should be interpreted with caution due to limited number of pooled studies, heterogeneity in retinal detachment characteristics and variation in surgical and postoperative protocols.

Importantly, both prone and supine groups showed postoperative improvement in logMAR BCVA when analyzed separately indicating visual recovery occurred with both positioning strategies. The direct comparison of prone versus supine demonstrated a very small borderline difference in favor of prone positioning, but this was based on just 2 studies with very low certainty of evidence. Therefore, the evidence available does not support a clinically meaningful functional superiority of prone versus supine positioning in routine practice. This interpretation is consistent with previous studies reporting no significant difference in postoperative visual acuity based on postoperative positioning strategy [21].

Despite the benefits offered by prone positioning, this approach has some limitations. Studies have shown that being in a prone positioning may cause increased intraocular pressure and pose a considerable burden to the patient both physically and mentally [21]. Since anatomical success remains unchanged, the benefit versus risk ratio for strict prone positioning comes into question.

A considerable level of heterogeneity existed in multiple analyses, mainly for BCVA, where I² exceeded 70%. Heterogeneity probably arose due to differences in various clinical and methodological characteristics between studies included in the meta-analysis. Visual function improvement following pars plana vitrectomy treatment for rhegmatogenous retinal detachment depends on several parameters other than positioning, such as the initial BCVA, macular involvement, macular detachment duration, ratio of macula-off retinal detachments, follow-up period, tamponade used, surgical procedure performed, and severity of retinal tears. The differences in these parameters might explain the heterogeneity in part. However, while meta-regression could possibly be useful in investigating whether those factors impacted the BCVA outcomes, it could not be used due to insufficient numbers of studies per BCVA analysis as well as the lack of data on relevant covariates for the studies involved. In this case, meta-regression would yield inaccurate and untrustworthy results due to being underpowered. Thus, the pooled BCVA results should be treated with caution.

The findings suggest that strict prone positioning is not essential for achieving retinal reattachment following PPV for RRD. Flexible or individualized positioning strategies may be appropriate in many cases, particularly given the high anatomical success rates observed across different approaches. However, prone positioning may still offer benefits in reducing retinal displacement and related visual symptoms. Therefore, postoperative management should balance potential benefits with patient burden, emphasizing individualized decision-making based on retinal pathology, surgical factors, and patient tolerance and compliance.

Aside from the success rate of retinal reattachment surgery and BCVA, research interest has shifted in recent years to the issue of postoperative retinal displacement and visual quality following vitrectomy surgery for RRDs [22]. Retinal displacement can occur even when a patient achieves an anatomically successful reattachment surgery, which may contribute to the development of postoperative metamorphopsia, aniseikonia, and other binocular disturbances that affect patient-reported visual quality [23]. The phenomenon of retinal displacement may result from residual subretinal fluid, gravity-related retinal shifting, postoperative positioning, and tamponade properties [24]. Moreover, the integrity of the ellipsoid zone and external limiting membrane and foveal microstructural recovery identified on OCT have been found to influence postoperative vision recovery and functional prognosis [25]. Differences in the tamponade substances used during the surgery may impact retinal stabilization and postoperative subretinal fluid resorption, ultimately affecting retinal displacement and photoreceptor recovery [3]. It is necessary, therefore, to use multimodal imaging techniques and patient-reported visual results along with standard anatomic measures of surgical success when assessing the effects of postoperative positioning [26].

The present study has several limitations that should be considered when interpreting the results. First, the included studies had considerable clinical heterogeneity. The studies differed in terms of macular status, location and number of retinal breaks, proportion of inferior retinal breaks, tamponade agent, vitrectomy technique, adjunctive procedures, duration of postoperative positioning, duration of follow-up, and definition of anatomical and visual outcomes. This makes direct comparison between studies difficult as these factors may independently affect retinal reattachment and visual recovery.

Second, the postoperative positioning strategies were not consistently defined and applied across the studies. Terms such as prone, face-down, supine, face-up, support-the-break, adjustable, free, no-prone and log-roll positioning were inconsistently used. The terminology was standardized for the purpose of synthesis, but actual clinical protocols, duration of positioning, patient instructions, and allowed alternative positions varied across studies. In addition, adherence to positioning was not measured or reported, which may have influenced the treatment effects observed.

Third, the number of studies included in each quantitative analysis was limited. This limited the statistical power of the pooled analyses and prevented meaningful subgroup analyses based on clinically relevant factors such as macular status, inferior retinal breaks, tamponade agent, lens status, duration of positioning or retinal detachment complexity. Meta-regression was not possible due to the low number of studies and incomplete reporting of relevant covariates.

Fourth, there was heterogeneity in some analyses, especially those associated with BCVA. This may be related to differences in baseline visual acuity, macular involvement, timing of surgery, duration of detachment, follow-up period, surgical complexity, outcome measurement and reporting format. Therefore, the pooled BCVA findings should be interpreted with caution, especially since some analyses were descriptive pre–post analyses rather than direct comparisons between positioning strategies.

Fifth, the evidence base included both randomized and observational studies including retrospective cohorts and case series. Some studies were limited methodologically by risk of bias, non-randomized group allocation, potential confounding by indication, incomplete reporting and differences in eligibility criteria. These issues reduced the certainty of the evidence and limited the ability to draw strong causal conclusions.

Finally, the certainty of evidence was low for retinal reattachment rate and very low for BCVA comparison prone versus supine. Thus, the lack of a statistically significant difference in reattachment rate should not be taken as proof of equivalence between the two positioning strategies and the small borderline BCVA finding should not be taken as evidence of clinically meaningful functional superiority of prone positioning.

Considering these limitations, individual postoperative positioning should be considered a conservative clinical implication rather than a definitive recommendation. Current evidence does not support widespread abandonment of strict prone positioning for all patients. Instead, positioning decisions should be patient specific and consider the location of the retinal break, the tamponade agent, the status of the macula, intraoperative findings, surgical protocol, patient tolerance and ability to comply, and surgeon judgment. Randomized controlled trials with better design, standardized positioning protocols, consistent outcome definitions, adherence monitoring and longer follow-up are needed to provide higher-certainty evidence.

Future research should prioritize large, well-designed randomized trials with standardized positioning protocols. Greater emphasis is needed on patient-reported outcomes, including quality of life and adherence, as well as stratified analyses based on retinal characteristics and tamponade type. The use of advanced imaging modalities should also be expanded to better assess retinal displacement and its functional consequences.

## Conclusion

The available evidence in this systematic review and meta-analysis showed no statistically significant difference in the retinal reattachment rate between prone positioning and support-the-break positioning following pars plana vitrectomy in rhegmatogenous retinal detachment. However, this finding needs to be interpreted with caution because of the low certainty of evidence for the outcome reattachment rate, mainly due to heterogeneity and imprecision. Therefore, the lack of a statistically significant difference should not be interpreted as equivalence between postoperative positioning strategies.

In direct comparative analysis, a very small borderline difference in logMAR BCVA favored prone positioning compared with supine positioning for visual outcomes. However, certainty of evidence for this comparison was very low due to the small number of studies, small sample sizes, indirectness and imprecision. The clinical importance of this small difference is unknown, and the current evidence does not support a strong conclusion that prone positioning provides clinically meaningful functional superiority.

Visual improvement was noted in both the prone and supine positioning arms on descriptive pre–post analyses, but such results largely reflect postoperative recovery after retinal detachment repair and cannot demonstrate a positioning-specific effect. Postoperative positioning should not be a blanket rule for all patients in general. Instead, the position should be tailored according to the site of the retinal break, tamponade agent, surgical considerations, patient tolerance and surgeon judgement. Additional well-designed randomized controlled trials with standardized positioning protocols, consistent outcome definitions and longer follow-up are needed to provide higher-certainty evidence.

## Supplementary Information

Below is the link to the electronic supplementary material.

Supplementary Material 1

## Data Availability

No datasets were generated or analysed during the current study.
