# Supplementary material for: Impact of postoperative positioning on surgical and visual outcomes after pars plana vitrectomy for rhegmatogenous retinal detachment: a systematic review and meta-analysis
Source: Int J Retina Vitreous. 2026 Jun 25;12:122. doi: 10.1186/s40942-026-00890-7 (PMC13563755; doi:10.1186/s40942-026-00890-7)
Supplement: Supplementary file 1 [file 40942_2026_890_MOESM1_ESM.docx]

| **Database** | **Search Query** |
| --- | --- |
| PubMed | (("Rhegmatogenous Retinal Detachment"[Mesh] OR "rhegmatogenous retinal detachment" OR RRD OR retinal detachment) AND ("Vitrectomy"[Mesh] OR "pars plana vitrectomy" OR PPV OR vitrectomy) AND ("postoperative positioning" OR positioning OR "prone positioning" OR prone OR "face-down positioning" OR supine OR "supine positioning" OR "support-the-break" OR "support-the-break positioning" OR adjustable OR "free positioning") AND ("visual acuity"[Mesh] OR "best corrected visual acuity" OR BCVA OR retinal reattachment OR anatomical success)) |
| Scopus | TITLE-ABS-KEY((“rhegmatogenous retinal detachment” OR RRD OR “retinal detachment”) AND (“pars plana vitrectomy” OR PPV OR vitrectomy) AND (“postoperative positioning” OR positioning OR prone OR “prone positioning” OR “face-down positioning” OR supine OR “supine positioning” OR “support-the-break” OR adjustable OR “free positioning”) AND (“visual acuity” OR BCVA OR “retinal reattachment” OR “anatomical success”)) |
| Web of Science (WOS) | TS=((“rhegmatogenous retinal detachment” OR RRD OR “retinal detachment”) AND (“pars plana vitrectomy” OR PPV OR vitrectomy) AND (“postoperative positioning” OR positioning OR prone OR “prone positioning” OR “face-down positioning” OR supine OR “supine positioning” OR “support-the-break” OR adjustable OR “free positioning”) AND (“visual acuity” OR BCVA OR “retinal reattachment” OR “anatomical success”)) |

**Table S1:** Search strategy for each database.

| **Synthesis status / outcome** | **Studies contributing** | **Number of studies** |
| --- | --- | --- |
| Retinal reattachment-rate meta-analysis | Casswell et al. 2020; Lin et al. 2017; Chen et al. 2015 | 3 |
| Prone versus supine BCVA comparison | Peiretti et al. 2017; Otsuka et al. 2017 | 2 |
| Prone-arm-only BCVA analysis | Peiretti et al. 2017; Chen et al. 2015; Shiraki et al. 2018; Otsuka et al. 2017 | 4 |
| Supine-arm-only BCVA analysis | Abdelkader et al. 2020; Peiretti et al. 2017; Otsuka et al. 2017 | 3 |
| Qualitative synthesis only | Liang et al. 2023; Schawkat et al. 2019; Babel et al. 2024 | 3 |

**Table S2:** Studies contributing to each quantitative and qualitative synthesis.

| **Outcome** | **No. of studies / participants** | **Effect estimate** | **Risk of bias** | **Inconsistency** | **Indirectness** | **Imprecision** | **Publication bias** | **Overall certainty of evidence** |
| --- | --- | --- | --- | --- | --- | --- | --- | --- |
| Retinal reattachment rate: prone vs support-the-break positioning | 3 studies / 674 eyes | RR = 0.96, 95% CI 0.84–1.11; p = 0.231 | Serious | Serious | Not serious | Serious | Undetected* | Low |
| BCVA change: prone vs supine positioning | 2 studies / 90 eyes | MD = −0.07 logMAR, 95% CI −0.14 to 0.00; p = 0.049 | Serious | Not serious | Serious | Serious | Undetected* | Very low |

**Table S3:** GRADE certainty-of-evidence assessment for pooled outcomes. *Publication bias was not formally assessed because fewer than 10 studies were included in each pooled analysis.
